# BACE2 degradation is mediated by both the proteasome and lysosome pathways

**DOI:** 10.1186/s12860-020-00260-7

**Published:** 2020-03-11

**Authors:** Kaixin Qiu, Wenping Liang, Shuai Wang, Tingting Kong, Xin Wang, Chunyan Li, Zhe Wang, Yili Wu

**Affiliations:** 1 Cheeloo College of Medicine, Shandong University, 44 Wenhua West Road, LixiaDistrict, Jinan, Shandong China; 2 Shandong Collaborative Innovation Center for Diagnosis, Treatment and Behavioral Interventions of mental disorders, Institute of Mental Health, Jining Medical University, 133 Hehua Road, Taibaihu New District, Jining, 272067 Shandong China; 3 Shandong Key Laboratory of Behavioral Medicine, School of Mental Health, Jining Medical University, 133 Hehua Road, Taibaihu New District, Jining, Shandong China; 4 The National Clinical Research Center for Geriatric Disease, Xuanwu Hospital, Capital Medical University, Beijing, China; 5 Advanced Innovation Center for Human Brain Protection, Capital Medical University, Beijing, China

**Keywords:** BACE2: half-life, Proteasome pathway: lysosome pathway

## Abstract

**Background:**

Alzheimer’s disease is the most common neurodegenerative disease in the elderly. Amyloid-β protein (Aβ) is the major component of neuritic plaques which are the hallmark of AD pathology. β-site APP cleaving enzyme 1 (BACE1) is the major β-secretase contributing to Aβ generation. β-site APP-cleaving enzyme 2 (BACE2), the homolog of BACE1, might play a complex role in the pathogenesis of Alzheimer’s disease as it is not only a θ-secretase but also a conditional β-secretase. Dysregulation of BACE2 is observed in Alzheimer’s disease. However, the regulation of BACE2 is less studied compared with BACE1, including its degradation pathways. In this study, we investigated the turnover rates and degradation pathways of BACE2 in both neuronal cells and non-neuronal cells.

**Results:**

Both lysosomal inhibition and proteasomal inhibition cause a time- and dose-dependent increase of transiently overexpressed BACE2 in HEK293 cells. The half-life of transiently overexpressed BACE2 protein is approximately 6 h. Moreover, the half-life of endogenous BACE2 protein is approximately 4 h in both HEK293 cells and mouse primary cortical neurons. Furthermore, both lysosomal inhibition and proteasomal inhibition markedly increases endogenous BACE2 in HEK293 cells and mouse primary cortical neurons.

**Conclusions:**

This study demonstrates that BACE2 is degraded by both the proteasome and lysosome pathways in both neuronal and non-neuronal cells at endogenous level and in transient overexpression system. It indicates that BACE2 dysregulation might be mediated by the proteasomal and lysosomal impairment in Alzheimer’s disease. This study advances our understanding of the regulation of BACE2 and provides a potential mechanism of its dysregulation in Alzheimer’s disease.

## Background

Alzheimer’s disease (AD) is the most common neurodegenerative disease in the elderly. Amyloid-β protein (Aβ) is the major component of neuritic plaques which are the hallmark of AD pathology [1]. Deposition of Aβ is formed from amyloid-β precursor protein (APP) by sequential cleavage of β- and γ-secretase [2].β-site APP cleaving enzyme 1 (BACE1) is the major β-secretase contributing to Aβ generation.β-site APP-cleaving enzyme 2 (BACE2), the homolog of BACE1, is a θ-secretase, which cleaves APP at Phe20 site to yield a CTF with 80 amino acids (CTFθ or C80) contributing to the generation of a truncated Aβ [3, 4]. Moreover, BACE2 prevents neuronal apoptosis by cleaving a potassium channel at the surface of plasma membrane [5]. However, our recent study demonstrated that BACE2 can be converted into a β-secretase with comparable β-secretase activity to that of BACE1, implying that BACE2 could contribute to Aβ generation in AD [6].Consistently, increased BACE2 expression and activity is detected in neurons of AD brains [7]. Genetic data highly supports that BACE2 is associated with AD risk. For example, BACE2 haplotype associates with AD, while SNPs in BACE2 (e.g., rs2252576, rs2837990, rs7281733) predispose to early onset of AD in patients with Down syndrome [8, 9]. Recently, the association between a number of SNPs in BACE2 and AD was detected in APOE ε4 non-carriers, which might be mediated by altered BACE2 expression-mediated Aβ generation and clearance [10]. It indicates that dysregulation of BACE2 might contribute to the pathogenesis AD.

It is important to elucidate the regulation of BACE2 expression as BACE2 homeostasis is critical to maintain the physiological function and counteract the pathogenesis of AD. In addition to the transcriptional regulation, protein degradation does play an important role in BACE2 homeostasis [11]. The ubiquitin-proteasome pathway (UPS) and the autophagy-lysosome pathway (ALP) are two major pathways for protein degradation in eukaryotic cells [12, 13]. The impairment of the proteasome and lysomsome activity in AD has been reported in a number of studies, which might contribute to the dysregulation of BACE2 in AD [14]. However, the degradation of BACE2 remains elusive. To further elucidate the feature of BACE2 degradation, we investigated BACE2 degradation in both neuronal and non-neuronal cells. We found that both lysosomal inhibition and proteasomal inhibition cause the increase of transiently overexpressed BACE2 in HEK293 cells. Moreover, both lysosomal inhibition and proteasomal inhibition markedly increases endogenous BACE2 levels in HEK293 cells and mouse primary cortical neurons, indicating that BACE2 is degraded by both the proteasome pathway and lysosome pathway. This work advances our understanding of the regulation of BACE2 and provides a potential mechanism of its dysregulation in AD. It might provide a potential strategy for the treatment of AD by targeting the dysregulation of BACE2 in AD.

## Results

### The half-life of transiently overexpressed BACE2 is approximately 6 h in HEK293 cells

**Fig. 1:**
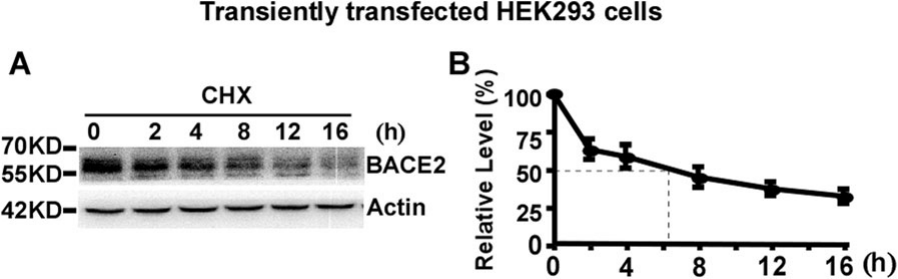
The half-life of transiently overexpressed BACE2 is approximately 6 h in HEK293 cells. **a** HEK293 cells were transfected with pBACE2-mychis. 48 h after transfection, cells were treated with 100 μg/mL CHX for 0, 2, 4, 8, 12 and 16 h, respectively. Cell lysates were resolved by 10% SDS-PAGE. BACE2 expression was detected by using 9E10. β-actin was detected by AC-15 and served as an internal reference. **b** Quantification of BACE2 levels at each time-point. BACE2 protein levels were plotted as a percentage of the amount of BACE2 level at 0 h. Values are mean ± SEM; *n* ≥ 3

Cycloheximide (CHX), also called actidione, was produced from Streptomyces griseus as a protein synthesis inhibitor [15].To examine the half-life of BACE2, HEK293 cells were transfected with plasmid pBACE2-mycHis. 24 h after transfection, cells were divided equally into six dishes. 48 h after transfection, the cells were treated with 100 μg/mL CHX to block BACE2 synthesis [16–18]. The cells were harvested at 0, 2, 4, 8, 12 and 16 h time-point, respectively. Western blot analysis was performed to measure the level of remaining BACE2 protein relative to the BACE2 level at 0 h time-point. BACE2 protein levels were decreased to 63 ± 6.6%, 58 ± 7.6%, 45 ± 6.7%, 37 ± 4.6% and 32 ± 4.7% at 2, 4, 8, 12 and 16 h time-point, respectively, *p* < 0.05 (Fig. 1a and b). Our data showed that the half-life of transiently overexpressed BACE2 is approximately 6 h.

### Lysosomal inhibition causes a time- and dose-dependent increase of transiently overexpressed BACE2 in HEK293 cells

**Fig. 2:**
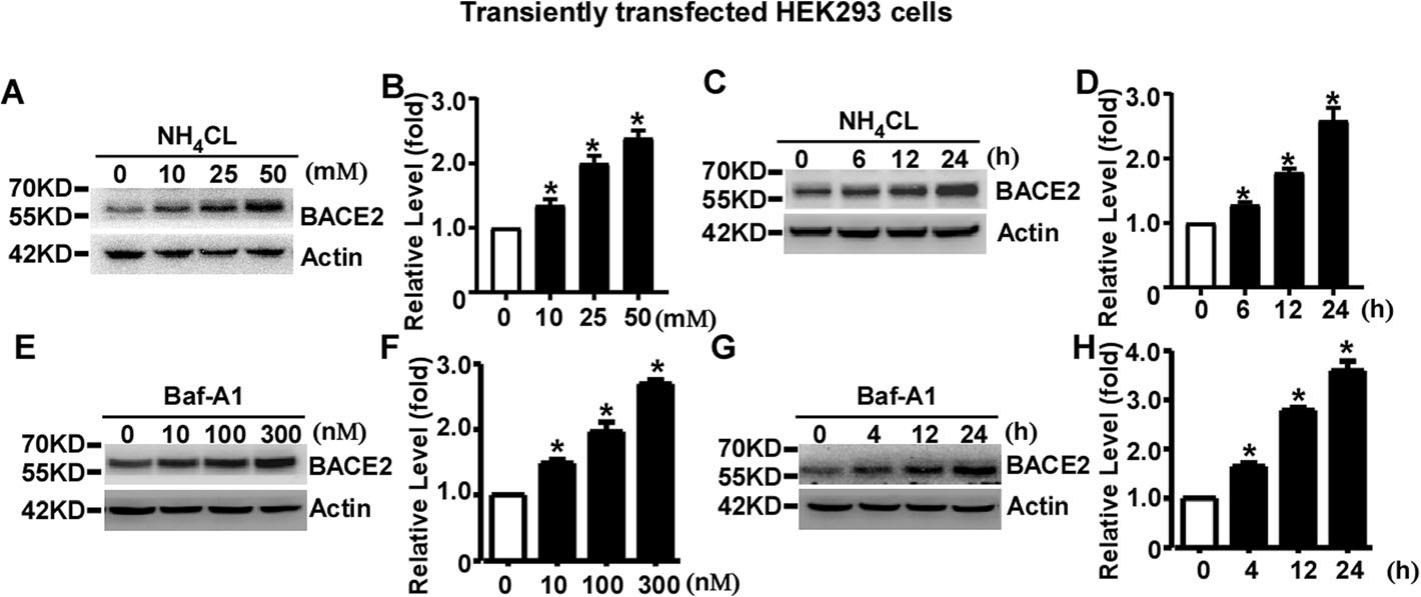
Lysosomal inhibition causes a time- and dose-dependent increase of transiently overexpressed BACE2 in HEK293 cells. **a** HEK293 cells were transfected with pBACE2-mychis. 48 h after transfection, cells were treated with 0, 10, 25 or 50 mM lysosome inhibitor NH_4_CL for 24 h. **b** Quantification of BACE2 levels. **c** HEK293 cells were transfected with pBACE2-mychis. 48 h after transfection, cells were treated with 25 mM NH_4_CL for 0, 6, 12 or 24 h. **d** Quantification of BACE2 levels. **e** HEK293 cells were transfected with pBACE2-mychis. 48 h after transfection, cells were treated with 0, 10, 100 or 300 nM Baf-A1 for 24 h. **f** Quantification of BACE2 levels. **g** HEK293 cells were transfected with pBACE2-mychis. 48 h after transfection, cells were treated with Baf-A1 for 0, 4, 12 or 24 h. (**h**) Quantification of BACE2 levels. 9E10 antibody was used to detect myc-tagged BACE2 protein. β-actin served as an internal control. Values represent mean ± SEM; *n* ≥ 3, *P < 0.05 by one-way ANOVA followed by Tukey’s test

To explore whether the lysosome pathway is implicated in BACE2 degradation, transfected HEK293 cells were treated with 0, 10, 25, or 50 mM of the lysosomal inhibitor NH_4_CL for 24 h. Western blot analysis showed that NH_4_CL treatment significantly increased BACE2 levels to1.35 ± 0.09, 2.00 ± 0.12 and 2.40 ± 0.12 fold, respectively, compared to that of control cells, *p* < 0.05 (Fig. 2a and b). For the time course assay, BACE2 were treated with 25 mM NH_4_CL for 0, 6, 12 and 24 h, respectively. The levels of BACE2 were significantly increased to 1.28 ± 0.04, 1.79 ± 0.06 and 2.60 ± 0.19 fold, respectively, *p* < 0.05 (Fig. 2c and d).

To further confirm the effect of lysosomal inhibition on BACE2 degradation, Bafilomycin A1(Baf-A1), an inhibitor of autophagosome-lysosome fusion, was applied to BACE2 transfected cells at 0, 10, 100 and 300 nM for 24 h, respectively. Baf-A1 treatment significantly increased BACE2 levels to1.49 ± 0.06, 1.97 ± 0.14 and 2.70 ± 0.06 fold, respectively, compared to that of control cells, *p* < 0.05 (Fig. 2e and f). For the time course assay, cells were treated with 100 nM Baf-A1 for 0, 4, 12 and 24 h, respectively. The levels of BACE2 were significantly increased to 1.65 ± 0.07, 2.79 ± 0.06 and 3.60 ± 0.19 fold, respectively, *p* < 0.05 (Fig. 2g and h).Our data showed that lysosomal inhibition causes a time- and dose-dependent increase of BACE2 expression, indicating that transiently overexpressed BACE2 is degraded by the lysosome pathway in HEK293 cells.

### Proteasomal inhibition increases transiently overexpressed BACE2 in HEK293 cells

**Fig. 3:**
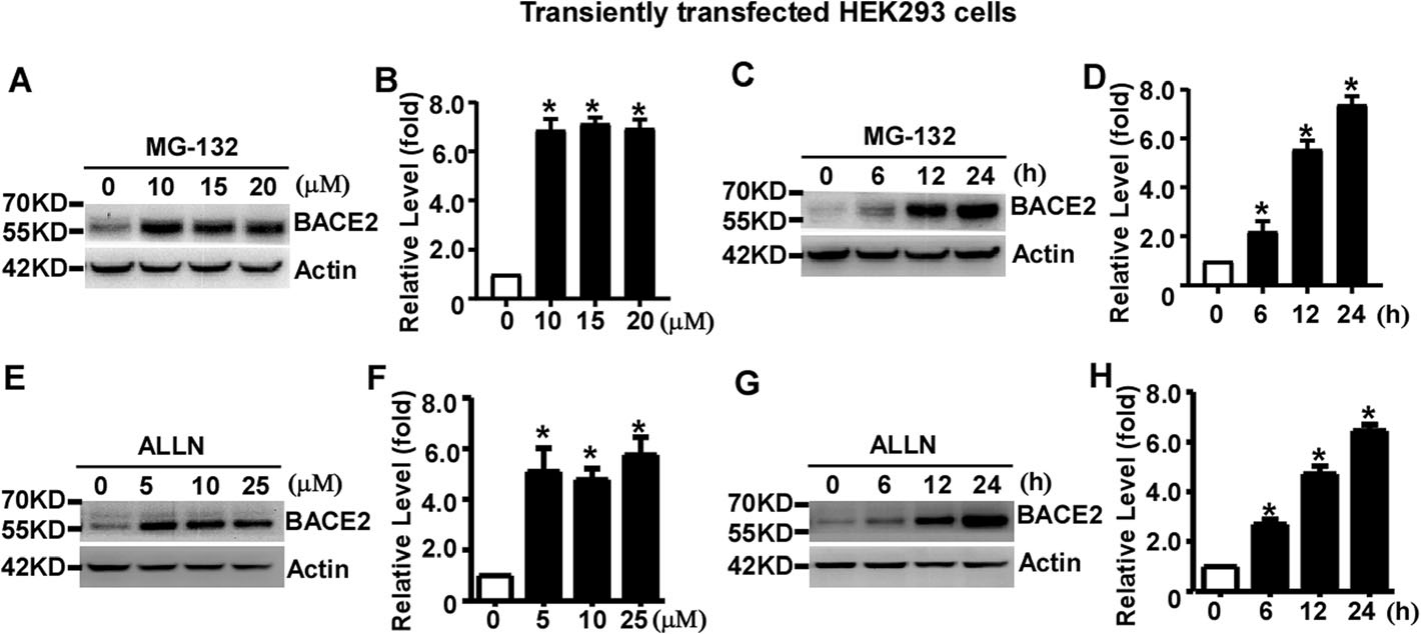
Proteasomal inhibition increases transiently overexpressed BACE2 in HEK293 cells. **a** HEK293 cells were transfected with pBACE2-mychis. 48 h after transfection, cells were treated with 0, 10, 15 or 20 μM proteasomal inhibitor MG-132 for 12 h. **b** Quantification of BACE2 protein levels. **c** HEK293 cells were transfected with pBACE2-mychis. 48 h after transfection, cells were treated with10 μM MG-132 for 0, 6, 12 or 24 h. **d** Quantification of BACE2 levels. **e** HEK293 cells were transfected with pBACE2-mychis. 48 h after transfection, cells were treated with 0, 5, 10 or 25 μM proteasomal inhibitor ALLN for 12 h. **f** Quantification of BACE2 protein levels. **g** HEK293 cells were transfected with pBACE2-mychis. 48 h after transfection, cells were treated with10 μM ALLN for 0, 6, 12 or 24 h. (**h**) Quantification of BACE2 levels. 9E10 antibody was used to detect myc-tagged BACE2 protein. β-actin served as an internal control. Values represent mean ± SEM; *n* ≥ 3, *P < 0.05 by one-way ANOVA followed by Tukey’s test

To explore whether the proteasome pathway is involved in BACE2 degradation, equal amount of pBACE2-mycHis transfected cells were treated with proteasomal inhibitor MG-132 at the concentration of 0, 10, 15 or 20 μM for 12 h. BACE2 levels were significantly increased to 6.87 ± 0.26, 7.14 ± 0.14 and 6.95 ± 0.20 fold at the dose of 10, 15 and 20 μM, compared to that in control cells, *p* < 0.05 (Fig. 3a and b). For the time course assay, cells were treated with 10 μM MG-132 for 0, 6, 12 and 24 h, respectively. The levels of BACE2 were significantly increased to 2.20 ± 0.24, 5.56 ± 0.20 and 7.40 ± 0.20 fold, respectively, *p* < 0.05 (Fig. 3c and d).

To further confirm the effect of proteasomal inhibition on BACE2 degradation, the proteasomal inhibitor ALLN was also applied to BACE2 transfected cells for 12 h. ALLN treatment significantly increased BACE2 levels to 5.107 ± 0.93, 4.78 ± 0.44 and 5.76 ± 0.71 fold at the dose of 5, 10 or 25 μM, respectively, compared to that in control cells, *p* < 0.05 (Fig. 3e and f). For the time course assay, cells were treated with 10 μM ALLN for 0, 6, 12 and 24 h. BACE2 levels were significantly increased to 2.69 ± 0.20, 4.72 ± 0.32 and 6.46 ± 0.25 fold, respectively, compared to that in control cells, *p* < 0.05 (Fig. 3g and h). It indicated that transiently overexpressed BACE2 is degraded by the proteasome pathway in HEK293 cells.

### The half-life of endogenous BACE2 is approximately 4 h in HEK293 cells

**Fig. 4:**
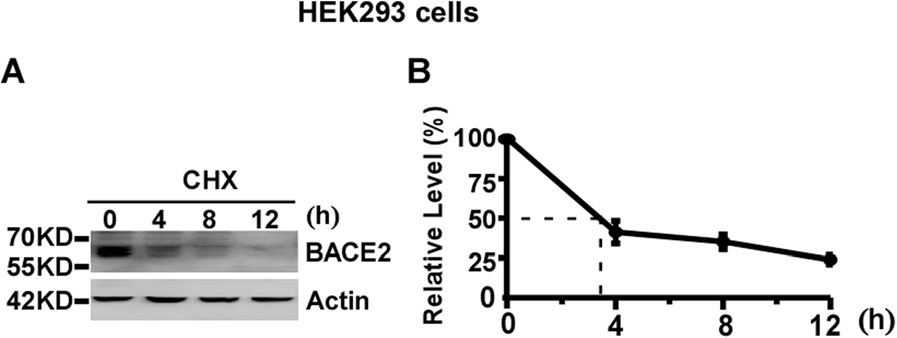
The half-life of endogenous BACE2 is approximately 4 h in HEK293 cells. **a** HEK293 cells were treated with 100 μg/mL CHX for 0, 4, 8 and 12 h, respectively. Cell lysates were resolved by 10% SDS-PAGE. BACE2 expression was detected by using anti-BACE2 antibody. β-actin was detected by AC-15 and served as an internal reference. **b** Quantification of BACE2 levels at each time-point. BACE2 protein levels were plotted as a percentage of the amount of BACE2 level at 0 h. Values are mean ± SEM; *n* ≥ 3

To examine the half-life of endogenous BACE2, HEK293 cells were divided equally into four dishes. The cells were treated with 100 μg/mL CHX to block BACE2 synthesis [16–18]. The cells were harvested at 0, 4, 8 and 12 h time-point, respectively. Western blot analysis was performed to measure the level of remaining BACE2 protein relative to the BACE2 level at 0 h time-point. BACE2 protein levels were decreased to 41 ± 7.0%, 35 ± 4.6% and 24 ± 2.9% at 4, 8 and 12 h time-point, respectively, *p* < 0.05 (Fig. 4a and b). Our data showed that the half-life of endogenous BACE2 is approximately 4 h in HEK293 cells.

### Both lysosomal inhibition and proteasomal inhibition increases the expression of endogenous BACE2 in HEK293 cells

**Fig. 5:**
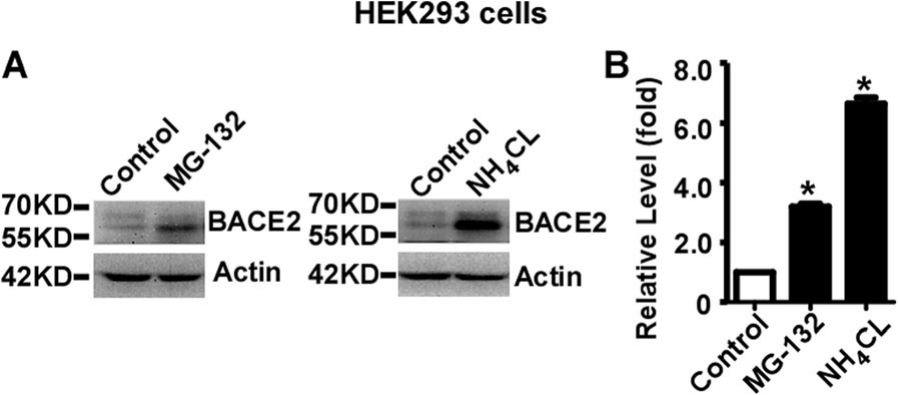
Both lysosomal inhibition and proteasomal inhibition increases the expression of endogenous BACE2 in HEK293 cells. **a** HEK293 cells were treated with vehicle (Control) and NH_4_Cl for 24 h, and MG-132 for 12 h, respectively. Anti-BACE2 antibody was used to detect endogenous BACE2 protein. β-actin served as an internal control and was detected by AC-15 antibody. **b** Quantification of BACE2 protein levels. Values represent mean ± SEM; *n* ≥ 3, *P < 0.05 by one-way ANOVA followed by Tukey’s test

HEK293 cells were divided equally into 6 cm dishes and treated with 10 uM MG-132 for 12 h and 25 mM NH_4_CL for 24 h, respectively. Endogenous BACE2 levels were significantly increased to 3.21 ± 0.10 and 6.65 ± 0.20 fold by MG-132 and NH_4_CL treatments, respectively, compared to that in control cells, *p* < 0.05 (Fig. 5a and b). Our data demonstrated that endogenous BACE2 is degraded by both the lysosome pathway and the proteasome pathway in HEK293 cells.

### The half-life of endogenous BACE2 is approximately 4 h in primary neurons

**Fig. 6:**
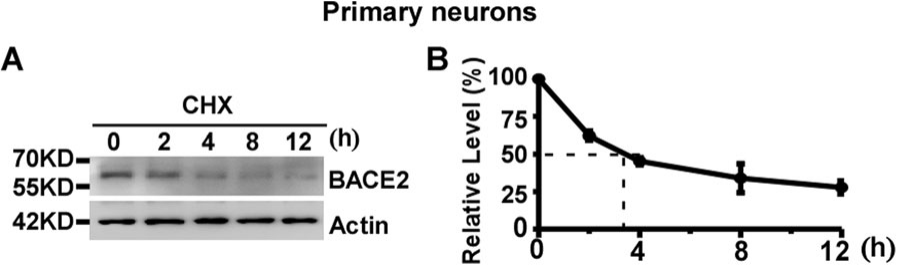
The half-life of endogenous BACE2 is approximately 4 h in primary neurons. **a** Primary cortical neurons were treated with 100 μg/mL CHX for 0, 2, 4, 8 and 12 h, respectively. Cell lysates were resolved by 10% SDS-PAGE. BACE2 expression was detected by using anti-BACE2 antibody. β-actin was detected by AC-15 and served as an internal reference. **b** Quantification of BACE2 levels at each time-point. BACE2 protein levels were plotted as a percentage of the amount of BACE2 level at 0 h. Values are mean ± SEM; *n* ≥ 3

To examine the half-life of endogenous BACE2 in primary neurons, mouse primary cortical neurons were isolated and seeded equally into five dishes. 7 days later, the cells were treated with 100 μg/mL CHX to block BACE2 synthesis [16–18]. The cells were harvested at 0, 2, 4, 8 and 12 h time-point, respectively. Western blot analysis was performed to measure the level of remaining BACE2 protein relative to the BACE2 level at 0 h time-point. BACE2 protein levels were decreased to 62 ± 2.9%, 45 ± 2.3%, 34 ± 9.6% and 28 ± 4.4% at 2, 4, 8 and 12 h time-point, respectively, *p* < 0.05 (Fig. 6a and b). Our data showed that the half-life of endogenous BACE2 is approximately 4 h in primary neurons.

### Both lysosomal inhibition and proteasomal inhibition increases the expression of endogenous BACE2 in primary neurons

**Fig. 7:**
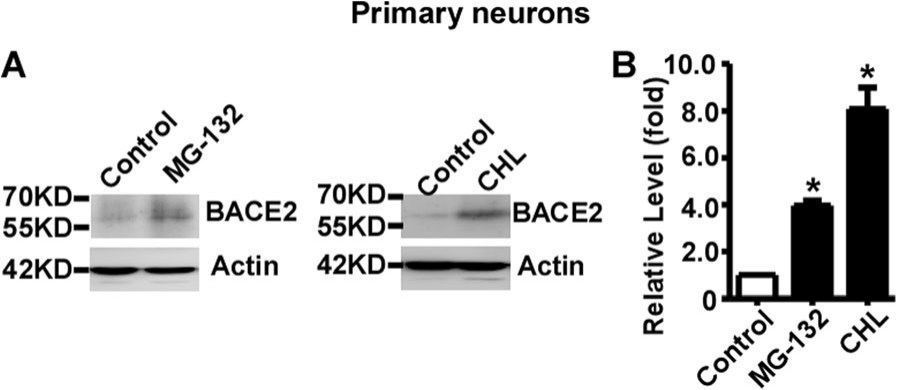
Both lysosomal inhibition and proteasomal inhibition increases the expression of endogenous BACE2 in primary neurons. **a** Mouse primary cortical neurons were treated with vehicle (Control) and CHL for 24 h, and MG-132 for 12 h, respectively. Anti-BACE2 antibody was used to detect endogenous BACE2 protein. β-actin served as an internal control and was detected by AC-15 antibody. **b** Quantification of BACE2 protein levels. Values represent mean ± SEM; *n* ≥ 3, *P < 0.05 by one-way ANOVA followed by Tukey’s test

The mouse primary cortical neurons were isolated and equally seeded. 7 days later, the primary neurons were treated with 100 μg/ml CHL and 5 μM MG132, respectively. Endogenous BACE2 levels were significantly increased to 3.97 ± 0.21 and 8.09 ± 0.90 fold by MG-132 and CHL treatments, respectively, compared to that in control cells, *p* < 0.05 (Fig. 7a and b). Our data demonstrated that endogenous BACE2 is degraded by both the lysosome pathway and the proteasome pathway in mouse primary cortical neurons.

## Discussion

The impairment of proteasome and lysosome function is reported in AD, and the dysregulation of BACE2 is also observed in AD. It suggests that impaired protein degradation function might play an important role in BACE2 dysregulation in AD by inhibiting BACE2 degradation. However, the feature of BACE2 degradation remains elusive. Thus, it is critical to elucidate the feature of BACE2 degradation. Both BACE1 and BACE2 are transmembrane proteins, and their amino acid sequences are 75% homologous. It is possible that BACE2 degradation might behave similarly to BACE1 which is degraded by both the proteasome and lysosome pathways [3, 19]. However, our previous study showed that BACE2 is only degraded by the autophagy-lysosome pathway in HEK293 cells stably overexpressing BACE2, but not by the proteasome pathway [18].

To further complement previous work performed in stable cells, we investigated BACE2 degradation at endogenous levels and under transient overexpression condition in both neuronal cells and non-neuronal cells. We found that both the lysosome and the proteasome pathways are implicated in the degradation of BACE2.The different conclusion drawn from the current study and the previous study might be caused by the following reasons. First, the clonal effect of stable cells might contribute to the difference as it might not represent the features of BACE2 in the population of cells. In addition, the high expression level and long-term overexpression might cause stronger stress on the cells in stable overexpression system. Moreover, long-term overexpression of BACE2 might have global effect on gene transcription including genes involved in protein degradation system, which inversely affects BACE2 degradation. For example, our previous study did show that long-term overexpression of transmembrane protein APP reduces proteasome activity by downregulating the expression of core subunits of the proteasome complex [20]. Furthermore, long-term protein overexpression might exhaust the capacity of the proteasome degradation system, resulting in proteasome impairment, thus, the proteasomal inhibitor has no effect on the proteasome-mediated BACE2 degradation in stable cells.

Our previous study showed that the half-life of stably overexpressed BACE2 is around 20 h [18]. In the current study, we showed that the half-life of transient overexpressed BACE2 is approximately 6 h, while the half-life of endogenous BACE2 is approximately 4 h. The dramatic difference between the half-life of stably overexpressed BACE2 and the half-life of transiently overexpressed BACE2 or endogenous BACE2 might be caused by the following reasons. First, the clonal effect of stable cells might contribute to the difference as it might not represent the features of BACE2 in the population of cells. Moreover, the relative long half-life of BACE2 in stable cells might be attribute to the impairment of proteasome pathway or the reduced proteasome activity as described above.

## Conclusions

In conclusion, we found that BACE2 is degraded by both the proteasome and the lysosome pathways in both neuronal cells and non-neuronal cells at endogenous level or in transient overexpression system, indicating that BACE2 degradation by both the proteasome and lysosome pathways is a common feature of BACE2. Moreover, the impairment of the proteasome pathway and the lysosome pathway in AD might lead to BACE2 dysregulation in neurons, contributing to the pathogenesis of AD [10]. This study not only advances our understanding of the regulation of BACE2 but also provides a potential mechanism of its dysregulation in AD. Moreover, it might provide a strategy for the treatment of AD by targeting the dysregulation of BACE2.

## Methods

### Cell culture and transfection

Human embryonic kidney HEK293 cells obtained from Dr. Weihong Song’s lab were cultured in high-glucose DMEM containing 10% fetal bovine serum and 1% penicillin-streptomycin. C57BL/6 mice were obtained from Cyagen Bioscience. Mouse cortical neurons were prepared from E18 embryos as described previously [6]. Briefly, the cortices of embryos were dissected out and the meninges were completely removed. The cortices were digested with papain at 37 °C for 20 min. Then, the papain solution was replaced with inactivation solution (MEM containing 0.6% D-[+]-glucose, 1 mM pyruvate, 10% horse serum, 2.5% bovine serum albumin [BSA], and 2.5% trypsin inhibitor) and the cells were dissociated by repeatedly pipetting. The isolated cells were equally seeded on poly-D-lysine–coated dishes with Neurobasal Media (Invitrogen) containing B27 [6]. All cells were maintained at 37 °C with 5% CO_2_ in an incubator as described previously [16, 18, 21]. pBACE2-mycHis refers to pZ-BACE2mycHis in this study, which is constructed previously [22]. Transient transfection was performed by using polyetherimide (PEI) method as described previously [23, 24]. Briefly, HEK293 cells were seeded 24 h prior to transfection. The regular culture medium was replaced with high-glucose DMEM without serum 1 h prior to transfection. 6 h after transfection, the medium was replaced with regular culture medium.

### Pharmacological treatment

HEK293 cells were transiently transfected with pBACE2-mycHis. 24 h after transfection, the cells were equally seeded into 6 cm culture dishes. 48 h after transfection, the cells were treated with different drugs, respectively. To measure the half-life of BACE2, 100 μg/mL cycloheximide (CHX) was used to treat cells as described previously [16, 18, 21]. After treatment, the cells were harvested at 0, 2, 4, 8, 12 and 16 h time-point, respectively. Lysosomal inhibitor NH_4_Cl, Bafilomycin A1(Baf-A1) and chloroquine (CHL) were applied to determine the involvement of lysosome pathway in BACE2 degradation, while proteasomal inhibitors N-carbobenzoxyl-L-leucinyl-L-leucinyl-L-leucinal (MG-132) and N-Acetyl-L-leucyl-L-leucyl-L-norleucinal (ALLN) were applied to determine the involvement of proteasome pathway in BACE2 degradation, respectively [16–18, 25, 26]. CHX, CHL and MG-132 were added to the primary cortical neurons at DIV7. MG-132, ALLN, NH4Cl, CHL, Baf-A1 and CHX were purchased from Sigma.

### Western blotting

Cells were lysed with RIPA-Doc buffer (Tris-HCl, 50 mM; NaCl, 150 mM; Triton X-100, 1%; deoxycholate, 1%; and SDS, 0.1%; supplemented with 1/100 protease inhibitors). Cell lysates were separated by 10% Tris-glycine SDS-PAGE gels and transferred to PVDF membranes. The membranes were blocked in 5% non-fat milk for 1 h, then incubated overnight at 4 °C with anti-myc (9E10), anti-BACE2 and anti-β-actin (AC-15) antibodies, respectively [18]. The membranes were washed in TBST with 0.1% Tween-20 and incubated with HRP-labeled goat anti-mouse or HRP-labelled goat anti-rabbit antibodies at room temperature for 1 h. Anti-myc antibody 9E10 was obtained from Abcam. Anti-BACE2 antibody was purchased from Santa Cruz (sc-271,212). β-actin antibody AC-15, HRP-labelled goat anti-rabbit antibody and HRP-labelled goat anti-mouse antibody were obtained from ZSGB-BIO. The image was obtained by using FluorChem R imaging system.

### Statistical analysis

The proteins expression was quantified by using ImageJ. One-way ANOVA followed by Tukey’s test was used for data analysis with three or more independent experiments. *P* < 0.05 was considered as a significant difference.

## Data Availability

The datasets used and/or analyzed during the current study are available from the corresponding author on reasonable request.

## Abbreviations

Aβ: Amyloid-β protein
AD: Alzheimer’s Disease
APP: Amyloid-β precursor protein
BACE1: β-site APP cleaving enzyme 1
BACE2: β-site APP cleaving enzyme 2
